# In vitro evaluation of some mechanical properties and fluoride release of glass-ionomer cement modified with seashell nanoparticles

**DOI:** 10.34172/joddd.41084

**Published:** 2024-09-07

**Authors:** Ahmed S. Albasso, Reem R. Ali, Abeer A. Yahya

**Affiliations:** ^1^Department of Pedodontics, Orthodontics and Preventive Dentistry, College of Dentistry, University of Mosul, Mosul, Iraq; ^2^College of Dentistry, Ibn Sina University For Medical and Pharmaceutical Sciences, Baghdad, Iraq

**Keywords:** Biomaterials, Minimally invasive dentistry, Seashell nanoparticles

## Abstract

**Background.:**

This research assessed the compressive strength and microhardness of glass-ionomer cement (GIC) after incorporating seashell nanoparticles and evaluated the inert fluoride-releasing ability.

**Methods.:**

Seashell nanoparticles were synthesized by a mechanical grinding protocol. These particles were characterized by transmission electron microscopy and energy dispersive x-ray and added to the glass-ionomer powder in a weight-to-weight ratio. Seventy-five study samples were distributed into eight samples for each study group (control, 5%, and 10% seashell) to have 24 samples for each test of the study (compressive strength, microhardness, and fluoride release). One sample per group was prepared for the Fourier transform infrared spectroscopy (FTIR) test. The fluoride ion release was measured after one and four weeks of incubation period at 37 ºC, while other tests were conducted after 24 hours of incubation.

**Results.:**

In all the test variables, the 10% seashell group showed the highest significant mean, followed by the 5% seashell and the control group. However, there was no significant difference between the 5% and 10% seashell groups in the first week of fluoride release.

**Conclusion.:**

According to the results, 10% seashell nanoparticles were the best to improve the mechanical properties of GIC and boost the fluoride-releasing potential.

## Introduction

 In developing countries, the atraumatic restorative technique (ART) was introduced to deliver instant caries management in remote rural areas where a specified treatment may not be available or affordable. ART restorations were performed by hand instruments and high-viscosity glass-ionomer cement (GIC).^1^

 GIC is extensively used in dentistry, such as luting cement, temporary restoration, and adhering orthodontic bands. ^2^ It is considered ideal for restoring dental caries according to the minimally invasive protocol, including ART.^3^ GIC confers exclusive preventive and anti-cariogenic properties due to its inert fluoride-releasing and recharging potential. It chemically adheres to dental tissues and has a coefficient of thermal expansion close to the tooth structure and reduced moisture sensitivity.^4^ Despite all these advantages, weak mechanical properties such as fracture resistance, compressive strength, and wear resistance are still the main weak points that compromise the durability of GIC, especially in restorations subject to high occlusal forces.^5^

 Nanoparticles have been demonstrated to improve the mechanical properties of dental materials. Therefore, the application of nanoparticles in the medical field has attracted researchers’ attention.^6^ Seashell nano-powder is considered a natural source of calcium ions. Most seashell powder is calcium carbonate in addition to phosphate, manganese, zinc, and other minerals.^7^ Seashells and eggshells, in addition to other biological sources like cuttlefish bone, are considered a natural origin to extract nano-hydroxyapatite, which has superior biocompatibility and is economically feasible.^8^ The incorporation of silica, hydroxyapatite, fluorapatite, and oyster shell nanoparticles, as well as resin, have been studied by many researchers as an attempt to reinforce the mechanical properties of traditional GIC and to boost the unique property of fluoride release.^9-11^ Hence, this study aimed to assess the mechanical properties of GIC after adding seashell nanoparticles, such as compressive strength and microhardness, and evaluate the fluoride-releasing inert ability.

## Methods

###  Materials 

 Seashell nanoparticle powder (prepared at the College of Dentistry, University of Mosul) was used in this study, in addition to Medifil, which is a radiopaque glass-ionomer restorative material (shade A2) from PROMEDICA Dental Material GmbH, Germany. The package contained 15 g of powder, 10 mL of liquid, and a spoon, with an expiry date of 2026.

###  Procedural steps

**Figure 1:**
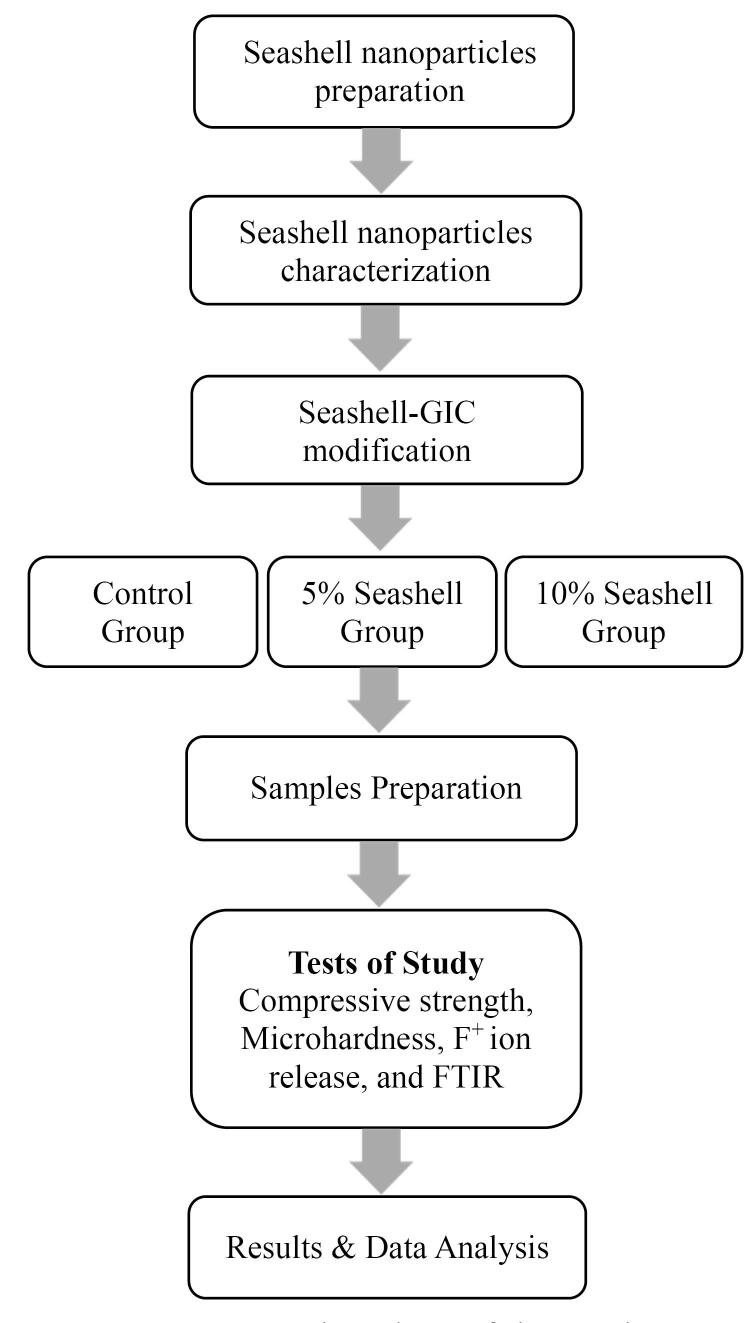
Flowchart of the study

 This research was approved by the Research Ethics Committee at the College of Dentistry, University of Mosul (reference no. UoM. Dent. 23/11). The methodological sequence of this study is described in Figure 1.

###  Preparation of seashell nanoparticles 

 Snail seashells used in this study were bought from the local market in Mosul city. Seashell nanoparticles were manually prepared using a mechanical grinding method according to the procedure described by Aidaros and Kamh.^12^ In this technique, 100 g of snail seashells were cleaned with water, boiled at 100 ºC for 30 minutes, and placed in the oven at 110 ºC for 2 days until dried. Afterward, the dried seashells were hand-grounded to achieve white powder using agate mortar. Using a ball mill machine (planetary-ball-mill-pm-400), the grounded powder was milled for 10 hours at 350 rpm.

###  Characterization of seashell nanoparticles 

 Seashell nanoparticles were examined at the Chemistry Analysis Center, Baghdad, under a transmission electron microscope (TEM) (Zeiss, EM10C, 100Kv, Germany) to determine the particle’s shape and size. The average particle size was calculated by measuring 20 particles using ImageJ software for image analysis (1.52 v). Energy dispersive x-ray analysis (EDX) was tested to determine the elemental percentages of seashell nano-powder.

###  Modification of seashell nanoparticles-GIC 

 GIC powder was mixed with seashell nanoparticles in a wt:wt ratio using a digital balance sensitive to 0.0001g (KERN, Germany). To obtain 5% seashell-GIC modified powder, 0.75 g of seashell nanoparticles was added to 15 g of GIC powder. 1.5 g of seashell nanoparticles was added to 15 g of GIC powder to produce 10% seashell-GIC modified powder. The two powder components for each produced percentage were manually mixed and placed on a dental vibrator for 1 minute to achieve a uniform mixture.^13^ The weight percent of added seashell nanoparticles was determined according to a previous pilot study, which revealed the highest compressive strength.

###  Sample size and grouping

 Using G*Power software (version 3.1.9.2.), the sample size was calculated with data alpha error of 0.05, 0.95 power of the study, and 0.88 effect size. The effect size was calculated using the same software based on the microhardness means and standard deviation of a previous study by Allam and Abd El-Geleel.^14^ The estimated sample size was 24 specimens for each test of the study for compressive strength, microhardness, and fluoride release. Three additional samples were used for the Fourier transform infrared spectroscopy test (FTIR) (n = 1/group). The total sample size of the study was 75 samples. The study groups were the control group (conventional glass-ionomer restorative cement without modification, 5% seashell group (glass-ionomer restorative cement modified by 5% seashell nanoparticles), and 10% seashell group (glass-ionomer restorative cement modified by 10% seashell nanoparticles.

###  Samples preparation and tests of the study

 Seventy-five samples of the study for control, 5% seashell, and 10% seashell groups were prepared by the same operator to decrease variability in the mixing procedure between the researchers. The samples were prepared by mixing a 1:1 powder-liquid ratio for 20 seconds on a non-absorbable paper pad with a spatula at room temperature according to the manufacturer’s instructions.

###  Compressive strength test 

 Following ISO specifications 9917-1:2007, 24 samples (n = 8/group) (6 mm in height, 4 mm in diameter) were prepared using a Teflon mold measured by a digital caliper.^15^ The molds were placed on a smooth, flat surface, filled with the mixed GIC material by a spatula, and pressed from the top by a glass slab (200 g of weight) to standardize the exerted pressure, eliminate the air bubbles from the mixed material, and achieve a smooth surface after setting finally.^16^ After 1 hour, the samples were removed from the molds and incubated (NUVE EN 400, Turky) at 37 ºC and 100% humidity inside a plastic container containing 10 mL of deionized water.^4^ After the incubation period (24 hours), the specimens were placed between the plates of the universal testing device (Gester, China). The device exerted a gradually increasing force through the vertical axis of the sample with a crosshead speed of 0.5 mm/min until fracture. Compressive strength value was calculated using the maximum force applied (F) in Newton, and the specimen diameter (d) according to the equation CS = 4F/ᴫd^2^.^17^

###  Surface microhardness test

 Twenty-four samples (n = 8/group) (6 mm in diameter, 3 mm in height) were prepared using a Teflon mold,^18^ following the same procedures for GIC mixing, insertion in the Teflon mold, and application of standard pressure previously described. After 1 hour, the samples were dislodged from the molds and incubated at 37 ºC and 100% humidity inside a plastic container containing 10 mL of deionized water. After 24 hours of incubation, the surface microhardness was measured by Vicker’s microhardness tester (Wolpert, Germany) by applying a 500-g pressure for 10 s twice from the center of each sample.^19^ Vicker’s microhardness value (HV) was calculated according to the formula: HV = 1.854*F/d^2^ (F is the applied load in kilogram, and d is the average diagonal length of the imprint in millimeters).^20^

###  Fluoride ion release test

 Under ISO specification 19448:2018, 24 specimens (n = 8/group) (6 mm in diameter, 4 mm in height) were prepared using a Teflon mold,^21^ following the same procedures for GIC mixing, insertion in the Teflon mold, and application of standard pressure described above. Before setting, the same length of dental floss was inserted into the samples to suspend them in the deionized water. The samples were retrieved from the molds after 1 hour. For additional standardization, each sample was weighed (0.25 g ± 0.01) by a digital balance. Each sample was vertically suspended in 10 mL of deionized water in a tightly closed, scaled plastic container and then incubated at 37 ºC and 100% humidity. Fluoride ion release from each sample was measured in ppm after one and four weeks by fluoride ion-selective electrode (Eutech Instruments, Singapore). The device was recalibrated to a standard solution of 6.00, 8.00, 10.00, 12.00, 14.00, and 16.00 ppm fluoride ion.^22^

###  FTIR spectroscopy test

 One sample (1 mm in thickness, 6 mm in diameter) from the control group and each experimental group was prepared using the same procedure described for the previous tests of the study and then crushed to powder. The samples were examined by FTIR device (Alpha Bruker, Germany) with 400‒4000 cm^−1^ wave number and 4 cm^−1^ resolution at room temperature to identify the functional groups of the specimens.^23^

###  Statistical analysis

 The normal distribution of data was tested using the Shapiro-Wilk test. Some descriptive statistics like means, minimums, maximums, and standard deviations were calculated. One-way ANOVA was used to compare the differences between the study groups of each test. Paired-samples t-test was used to identify the differences between weeks 1 and 4 regarding fluoride release from each study group. A significant difference was defined at *P* ≤ 0.05.

## Results

###  Transmission electron microscopy 

**Figure 2:**
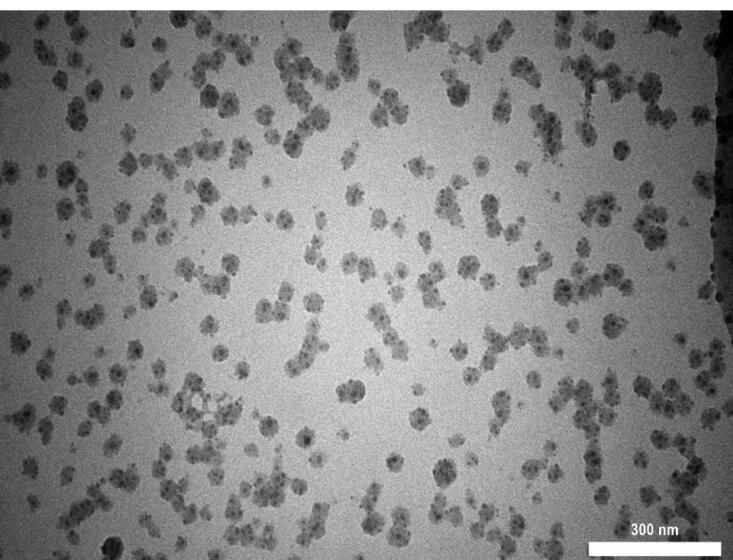
TEM image of seashell nanoparticles

 The TEM image of seashell nanoparticles revealed round, oval-shaped nanoparticles with an average particle size of 28.18 nm, as seen in Figure 2.

###  Energy dispersive X-ray 

 The element percentages of prepared seashell nanoparticles are presented in Table 1, which shows that calcium and phosphate ions have the highest element percentages at 20.10% and 23.75%, respectively.

**Table 1:** EDX analysis of seashell nanoparticles

**Elements**	**W%**	**A%**
C	8.21	14.06
O	46.14	59.30
P	23.75	15.77
Ca	20.10	10.31
Zn	1.79	0.56

###  Compressive Strength

Table 2 (section A) presents the statistics of compressive strength data for control, 5%, and 10% seashell groups. The highest mean of compressive strength (88.60 ± 1.05 MPa) was found in the 10% seashell group, while the control group showed the lowest mean (64.16 ± 0.94 MPa) with a significant difference (*P* = 0.000) between the three groups.

**Table 2:** Descriptive and analytic statistics of test variables

**Tests**	**Groups**	**Mean±SD (Min-Max)**
(A) Compressive strength	Control	67.41 ± 0.62 a (66.20 - 68.06)
	5% Seashell	69.01 ± 0.63 b (68.09 - 70.01)
	10% Seashell	93.09 ± 0.92 c (91.45 - 94.43)
	*P* = 0.000*	
(B) Surface microhardness	Control	49.96 ± 0.99 a (48.30 - 50.86)
	5% Seashell	53.41 ± 0.89 b (52.41 - 55.02)
	10% Seashell	55.72 ± 1.19 c (53.85 - 57.58)
	*P* = 0.000 *	
(C) Fluoride Release week 1	Control	7.10 ± 0.91 a (5.44 - 8.12)
	5% Seashell	9.18 ± 1.07 b (7.55 - 10.85)
	10% Seashell	10.35 ± 1.46 b (8.84 - 13.13)
	*P* = 0.000*	
(D) Fluoride Release week 4	Control	9.05 ± 1.85 a (6.33 - 11.37)
	5% Seashell	10.45 ± 1.01 b (9.09 - 11.96)
	10% Seashell	11.79 ± 0.34 c (11.41 - 12.24)
	*P* = 0.001	

SD: Standard deviation, Min: Minimum value, Max: Maximum value. * A highly significant difference; different small letters mean the significant differences between the groups of each test vertically.

###  Surface microhardness

Table 2 (section B) presents the statistics of surface microhardness data for the study groups. The 10% seashell group showed the highest microhardness mean (55.72 ± 1.19 MPa), followed by the 5% seashell group (53.41 ± 0.89 MPa) and the control group (49.96 ± 0.99 MPa), with a significant difference (*P* = 0.000) between the three groups.

###  Fluoride ion release

**Figure 3:**
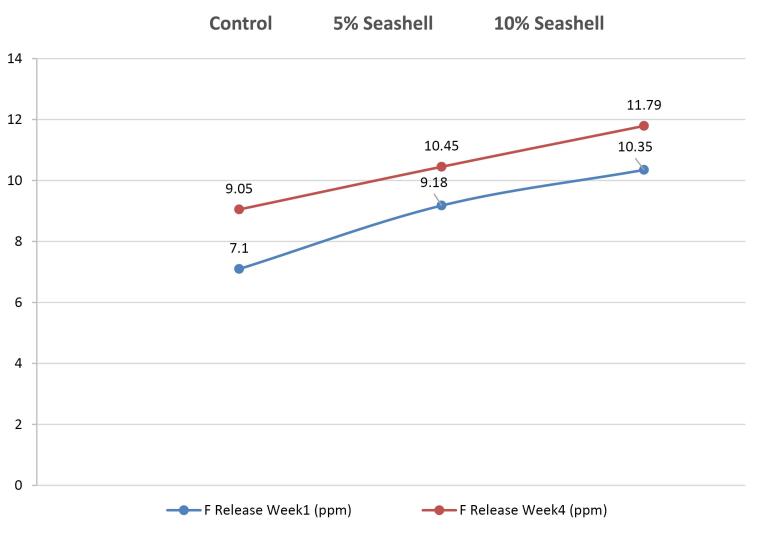
Fluoride release; week 1–week 4 comparison

 The statistical results of fluoride release in the first week for the control and experimental groups are illustrated in Table 2 (section c). The 10% seashell group had the highest fluoride release mean (10.35 ± 1.46 ppm), but it did not significantly differ from the 5% seashell group. The control group had the lowest mean (7.10 ± 0.91 ppm), with a significant difference between the control and the two seashell groups (*P* = 0.000). In the fourth week, the 10% seashell group also showed a higher mean (11.79 ± 0.34 ppm) than the 5% seashell group (10.45 ± 1.01), while the control group had the lowest mean (9.05 ± 1.85 ppm), with a significant difference (*P =*0.001) between the three groups (Table 2, section D). All the study groups exhibited a significant increase in fluoride release in the fourth week compared to the first week (*P =*0.046, *P* = 0.044, and *P* = 0.034 for each pair group, respectively) (Figure 3).

###  FTIR spectroscopy

**Figure 4:**
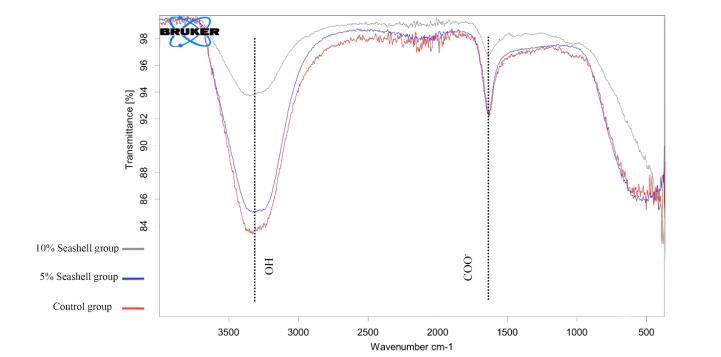
FTIR bands for the study groups

Figure 4 illustrates the appearance of the same broad bands at 3353, 3330, and 3354 cm^-1^ for control, 5%, and 10% seashell groups, respectively, which refer to O-H stretching. The same bands that nearly appeared at 1633 cm^-1^ indicated COO - vibration modes in the control, 5%, and 10% seashell groups.

## Discussion

 Regarding the minimal intervention criteria, which are a protocol of selective caries removal, GIC is required in this technique for restoration because it is a bioactive material that leads to the remineralization of demineralized dentin after caries removal.^24,25^

 Seashell nanoparticles in this study were synthesized from natural biological substances because of their biocompatibility, which had been referred to as non-toxic material by previous studies.^26^ In the current study, seashell nanoparticles were observed by TEM and EDX to identify the shape, size, and chemical components. TEM image showed round-oval-shaped nano-size particles. EDX analysis revealed a large amount of phosphate and calcium.

 Compressive strength and microhardness tests were selected in the mechanical properties assessment of GIC in this study because compressive strength simulates the masticatory force frequently applied to the restorative material in the oral cavity.^27^ Microhardness refers to materials that withstand plastic deformation.^28^ The present study’s results showed that 10% seashell nanoparticle’s addition to GIC could significantly increase both compressive strength and surface microhardness of GIC compared to 5% seashell, which was significantly higher than the control. Genaro et al^29^ reported that the round shape and nano size of particles ensured good distribution, increased surface area, and boosted the mechanical resistance as it efficiently filled the spaces within the ionomeric matrix, consistent with this research’s results and explaining the improved mechanical properties of GIC modified with 5% and 10% seashell nanoparticles.

 The current study’s results agree with those of Duarte et al,^27^ who found that calcium phosphate nanoparticles significantly increased the compressive strength of GIC. Effendi et al^30^ concluded that chicken-eggshell nano-hydroxyapatite significantly improved the surface hardness of the GIC. Alatawi et al^31^ found that different percentages of nano-hydroxyapatite significantly enhanced the compressive strength of GIC. In contrast, Ivanišević et al^32^ observed a significant reduction in the compressive strength of GIC modified with marine-derived hydroxyapatite micro powder. Bilić-Prcić et al^33^ reported reduced GIC microhardness values modified with micro-hydroxyapatite derived from cuttlefish bone except for the Fuji II 10 %, which might be due to the large size of microparticles, as they discussed.

 The ion-selective electrode test was used because it is accessible, sensitive, and a universal standard.^34^ Deionized water was used to guarantee that the fluoride released would not be affected by other mineral components of the storage medium. Adding 5% and 10% seashell nanoparticles significantly enhanced the fluoride release of GIC in the first and fourth weeks of incubation compared to the control group, as the highest release potential was in the 10% seashell group, followed by the 5% seashell group at both periods. The maximum fluoride release was due to the nanoparticle’s vast surface area, which increased acid-base interaction, elevating fluoride ion release.^8^ These results agree with those reported by Mahmoud and Metwally.^35^ These results are consistent with those of Nishanthine et al^36^; however, they concluded that incorporating chitosan nanoparticles improved the fluoride release property of GIC.

 FTIR is a spectroscopic analysis used to identify the structural composition of single and complex molecules. ^37^ It was used in the current study to explore the possible structural changes of GIC that may occur after modification with 5% and 10% seashell nanoparticles due to possible chemical reactions. The appearance of the same bands of OH and COO - functional groups in the control and experimental groups indicated that modifying GIC with 5% and 10% seashell nanoparticles did not induce any changes in the chemical structure, as no new bands appeared.

 The lack of the long-term mechanical resistance of seashell-modified GIC was one of the limitations of this study, in addition to the absence of the salivary constituents that are clinically present in the patient’s mouth. The interesting clinical relevance that would be investigated in future studies is the biological properties, calcium release, and re-mineralizing potential of seashell-modified GIC if used to restore deep carious lesions of teeth near the pulp.

## Conclusion

 Modifying GIC with 5% and 10% seashell nanoparticles improved mechanical properties like compressive strength and microhardness and boosted the fluoride-releasing ability after one and four weeks.

## Acknowledgments

 The authors appreciate the College of Dentistry, University of Mosul, for supporting this work.

## Competing Interests

 There were no conflicts of interest.

## Ethical Approval

 This research was approved by the Research Ethics Committee at the College of Dentistry, University of Mosul (reference no. UoM. Dent. 23/11) on 2/11/2023.
